# NSAIDs, Opioids, Cannabinoids and the Control of Pain by the Central Nervous System

**DOI:** 10.3390/ph3051335

**Published:** 2010-04-29

**Authors:** Horacio Vanegas, Enrique Vazquez, Victor Tortorici

**Affiliations:** Instituto Venezolano de Investigaciones Cientificas (IVIC), Apartado 20632, Caracas 1020A, Venezuela; E-Mails: enriquevr@yahoo.com (E.V.); victort@ivic.gob.ve (V.T.)

**Keywords:** NSAID, opioid, cannabinoid, descending pain control system, PAG, RVM

## Abstract

Nonsteroidal anti-inflammatory drugs (NSAIDs) act upon peripheral tissues and upon the central nervous system to produce analgesia. A major central target of NSAIDs is the descending pain control system. The rostral structures of the descending pain control system send impulses towards the spinal cord and regulate the transmission of pain messages. Key structures of the descending pain control system are the periaqueductal gray matter (PAG) and the rostral ventromedial region of the medulla (RVM), both of which are critical targets for endogenous opioids and opiate pharmaceuticals. NSAIDs also act upon PAG and RVM to produce analgesia and, if repeatedly administered, induce tolerance to themselves and cross-tolerance to opioids. Experimental evidence shows that this is due to an interaction of NSAIDs with endogenous opioids along the descending pain control system. Analgesia by NSAIDs along the descending pain control system also requires an activation of the CB1 endocannabinoid receptor. Several experimental approaches suggest that opioids, NSAIDs and cannabinoids in PAG and RVM cooperate to decrease GABAergic inhibition and thus enhance the descending flow of impulses that inhibit pain.

## 1. Introduction

The conscious experience of pain is one of the functional products of a set of neural structures in charge of detecting tissue damage and reacting to it. Tissue damage is detected by the distal terminals of peripheral neurons known as nociceptive primary afferents, which conduct this information up to their proximal axon terminals. These excitatory synaptic terminals are located in the dorsal horn of the spinal cord or in other neuronal groups in the central nervous system. Here information processing by second and further order nociceptive neurons, as well as by excitatory and inhibitory interneurons, may give rise to pain messages aimed at further targets. Some of these targets are neuronal circuits whose functional products are autonomic reflexes or somatic defensive reflexes and behaviors. Researchers purposely elicit in animals such defensive reflexes and more complex behaviors by applying standardized noxious stimuli to peripheral tissues, e.g., a hind paw, the colon, *etc.* The intensity of these reactions serves as an indicator of the degree to which pain messages are being transmitted and would eventually reach the brain and give rise to the conscious experience of pain. Another indicator is the discharge of action potentials by postsynaptic nociceptive neurons, e.g., in the spinal dorsal horn, when a standardized stimulus is applied to a peripheral tissue. Defensive reflexes and behaviors, and neuronal action potential discharges, are of course only surrogates of the real experience of pain, but they have greatly served to investigate the mechanisms of pain and the action of analgesics, so much so that frequently (if erroneously) increases in reflexes, behaviors or neuronal firing are referred to as “hyperalgesia” while their attenuation is referred to as “analgesia”.

Beyond the spinal cord, pain messages reach further targets along the ascending pain pathway—neuronal groups in the medulla, the pons, the midbrain, the hypothalamus and the thalamus—until the forebrain is reached—the amygdala and the insular, somatosensory and cingulate cortices. Here the neurons of the so-called “pain matrix” finally bring to consciousness the presence of damage in some specific somatic or visceral tissue.

This was pretty much the picture of the “pain system” until about 1970 [1]. Then three research lines fertilized each other and gave rise to the discovery of the opioid receptors, the endogenous opioids and the descending pain control system [2]. In the descending pain control system the nerve impulses flow from the forebrain to the spinal cord and other relay structures. In fact, at these structures the descending pain control system regulates the transmission of reflex and ascending messages, thus increasing or decreasing our sensitivity to pain caused by tissue damage. Whether the descending pain control system increases or decreases the transmission of pain messages depends on various circumstances. For example, in cases of primary inflammatory “hyperalgesia” the descending pain control system attenuates [3,4,5,6], whereas in cases of damage to a peripheral nerve the descending pain control system facilitates [7,8,9], the transmission of reflex and ascending pain messages [10].

One very important structure of the descending pain control system [11] is the gray substance located around the aqueduct of Sylvius in the midbrain, known as the periaqueductal gray matter (PAG). The PAG gathers information from several telencephalic—the somatosensory and cingulate cortices, the amygdala—and diencephalic structures—the thalamus, the hypothalamus—as well as from ascending pain pathways. Different regions of the PAG are involved in different functions. Regarding descending control of pain, the dorsal-dorsolateral portions of the PAG are involved in stress-induced analgesia, which is independent of opioids but depends on endocannabinoids [12]. The lateral-ventrolateral portions of the PAG are involved in opioid analgesia [11] and, as shall be exposed herein, in analgesia induced by NSAIDs. The PAG does not project to the spinal cord to any great extent; it rather funnels impulses onto the nucleus raphe magnus and neighboring structures of the rostral ventromedial medulla (RVM). In the RVM there are two classes of neuron that project to the spinal cord and whose involvement in pain control has been extensively documented: the on-cells, which facilitate, and the off-cells, which inhibit, transmission of pain signals [11].

Both the PAG and the RVM are well endowed with opioid receptors [13] and are thus greatly involved in the analgesic action of endogenous opioids and exogenous opiates [14,15,16,17,18,19,20]. Microinjection of morphine into the PAG of rats indirectly activates PAG output neurons and thereby causes a decrease in the activity of RVM on-cells and an increase in the activity of off-cells, thus simultaneously removing facilitation and increasing inhibition of spinal nociceptive neurons; this results in “analgesia”, that is, an attenuation of behavioral or spinal neuronal signs of nociception [11]. Microinjection of morphine into the RVM has similar effects. Systemic administration of opiates thus causes analgesia because, in addition to their direct action upon peripheral tissues and the spinal dorsal horn, they activate descending inhibition of pain messages by the descending pain control system.

The aim of the present review is to propose that non-opioid analgesics share common mechanisms for analgesia by the descending pain control system with endogenous opioids and, probably, endocannabinoids.

## 2. NSAIDs and Descending Inhibition of Pain

The analgesics known as nonsteroidal anti-inflammatory drugs (NSAIDs) have, with reason, traditionally been considered to be different from the opiates [21]. Yet well-known non-opioid analgesics and cyclooxygenase inhibitors such as diclofenac, aspirin and metamizol (dipyrone) [22,23,24,25], when microinjected into the PAG or the RVM of rats cause an inhibition of nocifensive reflexes [26,27,28,29]. Together with inhibition of nocifensive reflexes, when microinjected into the PAG or given systemically both aspirin and metamizol depress RVM on-cells and activate off-cells, as morphine does. Interestingly, the effect of diclofenac, aspirin or metamizol can be abolished by injecting naloxone, a broad spectrum opioid receptor antagonist, either systemically or directly into the PAG [26,30,31,32,33]. Although none of these NSAIDs is known to bind to opioid receptors, their action must be somehow related to the endogenous opioid system.

Inhibition of nocifensive reflexes in these experiments is interpreted as caused by inhibition of nociceptive neurons. But it might also be due to inhibition of motor neuronal circuits without alteration of ascending pain messages. Motor inhibition would have the unwanted consequence of leaving pain undiminished while preventing limb withdrawal from the noxious stimulus. Therefore, in order to be certain of an effect upon pain sensory pathways, action potentials from nociceptive neurons were recorded in the spinal dorsal horn of rats. Action potential discharges elicited by peripheral noxious stimuli applied to a hind paw were severely attenuated when metamizol was microinjected into the PAG [34]. Furthermore, when “hyperalgesia” was induced by inflaming a hind paw, PAG microinjection of metamizol also drastically attenuated dorsal horn neuronal responses to stimulation of the inflamed paw [35]. Again, this “analgesic” effect of PAG-microinjected metamizol was reversed when naloxone was administered to the PAG, the RVM or the spinal cord [32,36], thus suggesting that metamizol activates opioidergic circuits of the descending pain control system all the way from PAG down to spinal cord. These results reveal that analgesia by non-opioid analgesics involves a strong interaction with endogenous opioids in the descending pain control system. 

## 3. The Descending Pain Control System and NSAID-Induced Tolerance

Repeated administration of a drug can lead to a progressive loss of its effect. This is known as tolerance, and is particularly notorious for opiates. The PAG is crucial for tolerance to opiates [37,38,39,40,41]. The concept that endogenous opioids are somehow involved when NSAIDs act upon the descending pain control system to cause “analgesia” is supported by the finding in rats that microinjection of metamizol or aspirin twice daily into the PAG, or systemic administration of aspirin, ketorolac or xefocam twice daily, results in tolerance to the NSAID and cross-tolerance to morphine, whether microinjected into the PAG or given systemically [33,42,43]. Furthermore, an opioid-withdrawal syndrome can be triggered if naloxone is administered to metamizol- or aspirin-tolerant rats.

These findings were in agreement with the fact that tolerance to NSAIDs in humans had been suspected for a long time [44,45,46,47], and some clinical conditions, like medication overuse headache, can perhaps be interpreted as a withdrawal syndrome [48]. Also, systemic administration of diflunisal, a salicylic derivative, causes pharmocodynamic tolerance in rats [49], and ibuprofen, a well known NSAID, seems to induce tolerance in humans [50]. 

Tolerance to opiates is to some extent mediated by cholecystokinin, an endogenous peptide [51], and tolerance to systemic morphine administration can be prevented by systemic administration of cholecystokinin receptor antagonists [52,53,54,55]. One target of these effects is the PAG, because microinjection of proglumide, a nonselective cholecystokinin antagonist, into the PAG of rats prevents the development of tolerance to a subsequent microinjection of morphine into the same site [56]. Similarly, a microinjection of proglumide into the PAG of rats prevents the development of tolerance to a subsequent microinjection of metamizol into the same site [57]. Furthermore, a PAG microinjection of proglumide in metamizol- and morphine-tolerant rats restores the antinociceptive effect of a metamizol or morphine microinjection into the same site.

As already mentioned, the action of either opioid or non-opioid analgesics in the PAG leads to an excitation of PAG output neurons and this causes an activation of RVM off-cells and an inhibition of RVM on-cells, thus leading to “analgesia”. When tolerance develops, PAG microinjections of morphine [58] or metamizol [59] are no longer capable of affecting RVM neurons and inducing “analgesia”.

These results show further mechanistic relationships between opioid and non-opioid analgesics as regards the descending pain control system.

## 4. Are the Endocannabinoids Involved?

Cannabinoids are lipid molecules that activate metabotropic, G-protein coupled membrane receptors also activated by some derivatives of marihuana (*Cannabis sativa*). The best known cannabinoid receptor types are called CB1 and CB2, and both of them mediate inhibition of cellular processes [60,61]. The main endogenous ligands of CB1 and CB2 receptors, known as endocannabinoids, are arachidonoyl ethanolamide (anandamide, AEA) and 2-arachidonoyl glycerol (2-AG). The main metabolizing enzymes are fatty acid amide hydrolase (FAAH) for anandamide and monoacylglycerol lipase (MAGL) for 2-AG but also the cyclooxygenases can metabolize endocannabinoids [60,61]. The action of endocannabinoids upon their target cells is terminated by these enzymes and by cellular reuptake. GPR55 is a putative third cannabinoid receptor [62] and seems to be involved in hyperalgesia [63]. CB1 and CB2 receptors mediate the analgesic actions of cannabinoids.

Injection of cannabinoids into the cerebral ventricles [64], the lateral-ventrolateral PAG [65] or the RVM [66] induces “analgesia” in rats. Endocannabinoids in the dorsolateral PAG mediate stress-induced analgesia [12,67]. Cannabinoids in the RVM, whether exogenous or endogenous, activate off-cells and inhibit on-cells, giving rise to “analgesia”, and the RVM is critical for the “analgesic” effects of exogenous cannabinoids [68]. Cannabinoids are thus important mediators in the analgesic functions of the descending pain control system. 

An involvement of endocannabinoids in the analgesic effects of NSAIDs has been shown at a systemic level [69,70,71] and also locally in peripheral tissues [72,73]. The possibility that PAG endocannabinoids are involved in the analgesic effect of non-opioid analgesics was suggested by two lines of evidence. As already mentioned, microinjection of cannabinoids into the lateral-ventrolateral PAG causes “analgesia” [65]. This region is precisely where the metamizol, aspirin, morphine, naloxone and proglumide microinjections described above took place. On the other hand, the antinociceptive effects of non-opioid analgesics in the spinal cord can be prevented [74] or reversed [75] by AM251, an antagonist/reverse agonist of the CB1 receptor. This hints to a plausible relationship between cyclooxygenase inhibitors and endocannabinoids in analgesic mechanisms within the central nervous system. Would cannabinoid receptor antagonists block the “analgesic” effect of PAG-microinjected NSAIDs? Inflammation of a hind paw in rats leads to increased action potential discharges of nociceptive spinal neurons when the paw is stimulated. This “hyperalgesia” can be attenuated by a microinjection of metamizol into the PAG, as mentioned above [35]. Preliminary experiments now show that a subsequent microinjection of AM251 into the same PAG site or into the RVM reverses the “analgesic” effect of PAG metamizol [76]. This implies that PAG and RVM endocannabinoids and CB1 receptors are, as in the spinal cord, at least partly responsible for the local analgesic effect of NSAIDs.

## 5. Possible Mechanisms of Interaction

**Figure 1:**
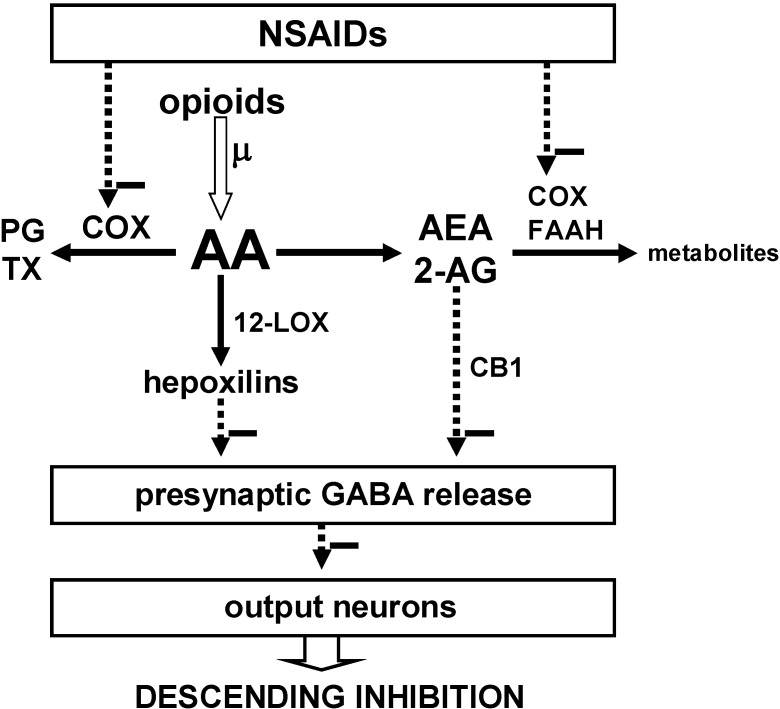
Proposed model for the interaction of NSAIDs, opioids and cannabinoids in the descending pain control system to induce analgesia. Minus symbols indicate inhibition. Inhibition of the cyclooxygenases (COX) by NSAIDs reduces the synthesis of prostaglandins (PG) and thromboxanes (TX) and thus increases the availability of arachidonic acid (AA). Opioids also increase the availability of AA by activating the phospholipase A_2_ via the µ-opioid receptor. Via the 12-lipoxygenases (12-LOX) AA is transformed into hepoxilins, which indirectly inhibit GABA release. By inhibiting COX and FAAH the NSAIDs spare AEA and 2-AG, which bind to the CB1 receptor (The role of the CB2 receptor in this model has not been established.) and thus inhibit GABA release. Removal of inhibition by GABA enhances the activity of output neurons that inhibit pain.

The most widely demonstrated mechanism for the analgesic action of NSAIDs is the inhibition of cyclooxygenases [22,23,24,25], but it has become increasingly obvious that this and other mechanisms might underlie an interaction between NSAIDs and other analgesics [21,77,78]. The bases for the interaction between NSAIDs, opioids and cannabinoids in the central nervous system and, more specifically, in the descending pain control system, are still poorly known, but several possibilities deserve consideration (Figure 1).

The fact that the action of NSAIDs in the PAG is related to opioids could be explained if NSAIDs increased the release of endogenous opioids, but this has never been investigated. Nonetheless there is experimental evidence for other types of interaction. Opioids in the PAG, by way of the µ receptor activate phospholipase A_2_ and thereby increase the availability of arachidonic acid [79]. Also NSAIDs, by blocking the cyclooxygenases, prevent the utilization of arachidonic acid for the synthesis of prostaglandins and thromboxanes and increase the availability of arachidonic acid for other molecular pathways. The 12-lipoxygenases then convert arachidonic acid into several compounds including the hepoxilins, which decrease the release of GABA from the synaptic terminals of inhibitory PAG axons [80]. Thus, increase in arachidonic acid availability leading to attenuation of synaptic inhibition is plausibly the mechanism where NSAIDs and opioids converge [81], and attenuation of GABAergic inhibition in the PAG causes “analgesia”. Indeed, microinjection of GABA antagonists into the PAG has the same “analgesic” effect as microinjection of morphine [82]. It thus seems that reduction of GABAergic inhibition increases the activity of PAG output neurons leading to “analgesia”. Also in the RVM a reduction of GABAergic inhibition has the same effect as morphine, *i.e.*, an increase in the activity of off-cells, which are output neurons that mediate spinal nociceptive inhibition [83]. This might also be one of the mechanisms of “analgesia” by RVM-microinjected NSAIDs [29].

As with opioids, one possible mechanism for the analgesic action of NSAIDs could be their induction of endocannabinoid release. Again, at least as far as the PAG and the RVM are concerned, this has not been investigated. Once more, a plausible link between NSAIDs and endocannabinoids may be related to the fact that NSAIDs inhibit the cyclooxygenases and the FAAH (Figure 1), and that these enzymes metabolize the endocannabinoids [60,78,84,85,86]. By inhibiting the cyclooxygenases and the FAAH, NSAIDs may therefore prevent enzymatic removal of endocannabinoids which, through the CB1 and CB2 receptors, induce analgesia, as mentioned above. For example, in the spinal cord a selective cyclooxygenase-2 inhibitor prevented rundown of 2-AG and caused “analgesia” during knee inflammation [75]. As a special case, acetaminophen may additionally be transformed into AM404, which boosts the action of anandamide by inhibiting FAAH and the cyclooxygenases and by blocking its cellular reuptake [71,78].

In the lateral-ventrolateral PAG and in the RVM, cannabinoids via the CB1 receptor inhibit the presynaptic release of GABA and thus enhance the activity of postsynaptic neurons, like opioids do [87,88]. Therefore, by preventing the removal of endocannabinoids, NSAIDs may facilitate the activity of descending neurons in charge of attenuating the transmission of pain signals.

As with all models, the model proposed to describe the interactions between NSAIDs, endogenous opioids and endocannabinoids (Figure 1) does not explain all experimental findings, and this has been discussed elsewhere [13]. For example, some PAG output neurons that project to RVM express µ-opioid receptors and would thus be postsynaptically inhibited by opioids. These neurons might still be presynaptically disinhibited by opioids, NSAIDs and cannabinoids and, if this disinhibition is greater than the postsynaptic inhibition, their activity would be nevertheless enhanced, as the model predicts. At any rate, the PAG-RVM neurons that are directly inhibited by opioids represent only 15% of the total. Another interesting finding is the inhibition of presynaptic glutamate release by µ-opioids and cannabinoids. This would decrease, not increase, the activity of postsynaptic neurons. How this finding fits the model cannot be inferred until the identity and function of the neurons involved can be characterized.

Thus, in spite of some uncertainties, the bulk of experimental evidence indicates that disinhibition by endogenous opioids and endocannabinoids of brain stem neurons that mediate descending inhibition of nociception [11,14] is the final common path for the analgesic effect of NSAIDs in the descending pain control system.

## 6. Conclusion

Experiments in laboratory animals have shown that NSAIDs, in addition to their actions at peripheral tissues and the spinal cord, exert their analgesic effects by activating the descending pain control system at the PAG and the RVM. Like the opioids and cannabinoids, NSAIDs act at the descending pain control system by activating RVM pain-inhibiting neurons and inhibiting RVM pain-facilitating neurons whose axons descend onto the spinal dorsal horn. The analgesic effects of NSAIDs at the PAG are at least partly related to endogenous opioids and cannabinoids and in the end indirectly result in an attenuation of GABAergic synapses, thus increasing the activity of output neurons responsible for descending inhibition. Repeated administration of NSAIDs progressively leads to tolerance to the NSAID, cross-tolerance to morphine and the risk of a withdrawal syndrome. These findings are important for human and animal medicine.
